# Family involvement, patient safety and suicide prevention in mental healthcare: ethnographic study

**DOI:** 10.1192/bjo.2023.26

**Published:** 2023-03-23

**Authors:** Louise S. Gorman, Donna L. Littlewood, Leah Quinlivan, Elizabeth Monaghan, Jonathan Smith, Stephen Barlow, Roger T. Webb, Navneet Kapur

**Affiliations:** National Institute for Health and Care Research (NIHR) Greater Manchester Patient Safety Translational Research Centre, University of Manchester, Manchester, UK; and Centre for Mental Health and Safety, School of Health Sciences, University of Manchester, Manchester, UK; National Institute for Health and Care Research (NIHR) Greater Manchester Patient Safety Translational Research Centre, University of Manchester, Manchester, UK; Centre for Mental Health and Safety, School of Health Sciences, University of Manchester, Manchester, UK; and Manchester Academic Health Science Centre, University of Manchester, Manchester, UK; National Institute for Health and Care Research (NIHR) Greater Manchester Patient Safety Translational Research Centre, University of Manchester, Manchester, UK; National Institute for Health and Care Research (NIHR) Greater Manchester Patient Safety Translational Research Centre, University of Manchester, Manchester, UK; Centre for Mental Health and Safety, School of Health Sciences, University of Manchester, Manchester, UK; Manchester Academic Health Science Centre, University of Manchester, Manchester, UK; and Greater Manchester Mental Health NHS Foundation Trust, Manchester, UK

**Keywords:** Family/carer involvement, self-harm, suicide, ethnography, qualitative research

## Abstract

**Background:**

Family involvement has been identified as a key aspect of clinical practice that may help to prevent suicide.

**Aims:**

To investigate how families can be effectively involved in supporting a patient accessing crisis mental health services.

**Method:**

A multi-site ethnographic investigation was undertaken with two crisis resolution home treatment teams in England. Data included 27 observations of clinical practice and interviews with 6 patients, 4 family members, and 13 healthcare professionals. Data were analysed using framework analysis.

**Results:**

Three overarching themes described how families and carers are involved in mental healthcare. Families played a key role in keeping patients safe by reducing access to means of self-harm. They also provided useful contextual information to healthcare professionals delivering the service. However, delivering a home-based service can be challenging in the absence of a supportive family environment or because of practical problems such as the lack of suitable private spaces within the home. At an organisational level, service design and delivery can be adjusted to promote family involvement.

**Conclusions:**

Findings from this study indicate that better communication and dissemination of safety and care plans, shared learning, signposting to carer groups and support for carers may facilitate better family involvement. Organisationally, offering flexible appointment times and alternative spaces for appointments may help improve services for patients.

Suicide is a leading cause of preventable death, with approximately 700 000 suicide deaths occurring worldwide each year.^1^ One key aspect of suicide prevention is the delivery of effective mental healthcare.^2^ A reduction in suicide has been reported by services that have implemented practice improvements, such as the removal of ligature points from hospital settings and timely follow-up contact post-discharge.^3,4^ Additionally, effective family involvement has been highlighted as a key area of practice that may help to prevent suicide,^5,6^ improve clinical and psychosocial outcomes for patients^7,8^ and support transitions in care.^9^

Reports of suboptimal family involvement and communication have been linked to compromised patient safety, as highlighted by findings from investigations into suicides and attempted suicides.^5,6,10,11^ Such studies have provided insight into what constitutes effective family involvement. In an analysis of clinicians’ views, effective family involvement was defined as establishing regular two-way communication and information sharing between families and healthcare services, responding to family members’ concerns and providing or signposting support for family members’ own health needs.^12^ A content analysis of coroners’ recommendations from suicide investigations indicated that including family members in the assessment process often helped to build a more comprehensive picture.^5^

Collectively, this evidence indicates that effective family involvement may improve patient outcomes. However, implementation of enhanced family involvement is inconsistent.^6,13,14^ The overarching aim of the current study was to investigate how families can be effectively involved in supporting a patient accessing services provided by crisis resolution and home treatment teams (CRHTTs) in England, using ethnographic methods.

## Method

### Study overview

Given that evidence suggests organisational context may act as a barrier to effective family involvement,^15^ a multi-site ethnographic approach was taken, encompassing multiple methods of data collection: (a) review of organisational documents in relation to family involvement, (b) observations of interactions between patients, family members and healthcare professionals, (c) case-note review in relation to family involvement and (d) semi-structured interviews with patients, family members and healthcare professionals.

### Study sampling and recruitment

#### Study setting

The study was conducted with two CRHTTs based in different National Health Service (NHS) mental health trusts in England. CRHTTs provide rapid assessment and intensive home treatment for people experiencing mental health crisis, as an alternative to hospital admission. The service model advises that CRHTTs should provide a single point of access and 24 h service.^16^ Some of the key features of CRHTTs have been described as managing patient care in the home environment and the involvement of family or carers throughout the care pathway;^17^ however, presence of support networks is not a prerequisite for home treatment. Trusts were approached according to their performance in meeting at least one of several quality criteria (a Care Quality Commission rating of ‘good’, low patient suicide rates, improved patient safety following service changes and provision of 24/7 access).

#### Participants

Participants comprised: (a) patients who were currently receiving care from a CRHTT, (b) members of their family/friends who were involved in their care and (c) healthcare professionals. Staff identified potential patient participants from their current case-load. Patient participants were eligible if they were adults aged 18 years or above who were currently accessing support from a CRHTT and from their partner, relative, friend or unpaid carer. All participants were able to provide written informed consent. Potential participants were excluded if they were unable to provide informed consent owing to the presence of gross cognitive impairment due to psychosis, intellectual disability, dementia, brain injury or intoxication. Patients who were unduly agitated or who were aggressive or threatening or who had history of violent behaviour were also excluded, as were individuals under the age of 18 years, as service provision is different for younger people in the UK. A maximum variation approach to sampling was taken,^18^ in which patients were recruited to cover different ages, genders and varying experiences of family involvement. Typically, the research sites have a case-load of 100–130 patients at any given time, equating to between 4 and 7 patients per keyworker plus any additional visits. To reduce the burden of the research on staff, staff were not asked to record the number of files considered or the percentage of patients who were approached.

#### Recruitment

Two lead post-doctoral researchers (L.S.G. and D.L.L.) held information sessions to invite staff at the research sites to participate in the study. Potential patient participants were identified in conjunction with healthcare staff, based on information contained in medical records. Initial contact with potential patient participants was made via healthcare teams to briefly introduce them to the study and ask whether they would like further information from the research team. Potential carer, family and friend participants were identified by approaching the support networks of patient participants.

#### Ethics

The authors assert that all procedures contributing to this work comply with the ethical standards of the relevant national and institutional committees on human experimentation and with the Helsinki Declaration of 1975, as revised in 2008. All procedures involving human subjects/patients were approved by the NHS Health Research Authority North West – Greater Manchester South Research Ethics Committee (reference: 19/NW/0294).

In considering the relational ethics that such sensitive research entails, we recognised the importance of building rapport and developing open and trusting relationships with participants. Therefore, observations were conducted first to allow patient and carer participants to become familiar with the researcher prior to participating in a one-to-one interview. Given that data were collected over multiple sessions, the researchers obtained written consent at the outset and periodically reconfirmed verbal consent with participants across the data collection period. Researchers worked closely with clinical leads, who facilitated relationships with staff participants and in turn staff participants facilitated relationships with patients and carers. Patient and carer participants chose where they would like to be interviewed, with two requesting a location outside of the home as they felt their home environment was not conducive to privacy. Researchers were flexible with interview locations and times to accommodate participants’ needs. Given that this study was focused on individuals accessing crisis mental healthcare, a safety protocol was included which specified that the researcher would break confidentiality should participants disclose any information that posed a risk to the health and safety of the participant or others. Both researchers sought support and received clinical supervision during the data collection phases. Further information regarding the ethical protocol and safety procedures is available from the corresponding author on request.

#### Patient and public involvement

Members of our patient and carer advisory panel were involved in all aspects of the research process, including the study design, interview guide development, conduct, reporting and dissemination plans for the research. Three panel members (who are also co-authors of this article: E.M., S.J.B, J.S.) with experience as patients or carers in this area contributed to the analytical framework and the development of emerging and final themes.

### Data collection

Data collection spanned the two services (site one and site two) and included 29 practice observations and interviews with 6 patients, 4 family members and 13 staff members (Table 1). Three potential participants expressed interest but did not take part in the study. Additional data were extracted from patients’ case notes, and the participating trusts’ policies referring to family involvement were reviewed. Data were collected across a 12-week period in 2019 at site one (before the COVID-19 pandemic) and in 2020–2021 (during the pandemic) at site two. Observations were carried out in the patient's home or a hospital setting and covered different time periods (morning, afternoon, evening, weekdays and weekends). Interviews were carried out face to face in the patient's home or via telephone and were audio-recorded and transcribed verbatim. Interviews lasted between 20 and 180 min. Data were gathered systematically. First, trust policies were reviewed, followed (where possible in the context of COVID-19 restrictions) by observations of clinical practice, allowing for specific areas of focus in the interviews. To protect anonymity, all participants were assigned a pseudonym which depicted their participant type (P, patient, F, family member, H, healthcare professional) and service (S1, site one; S2, site two).

**Table 1:** Overview of data collected at each research site

Site (researcher)	Duration	Observations	Interviews
One (D.L.L.)	12 weeks (2019)	20	12 (4 patients, 3 family members, 5 staff)
Two (L.S.G.)	21 weeks (2020–2021)	9	11 (2 patients, 1 family members, 8 staff)

### Analysis

An iterative-inductive, constant comparison approach was taken whereby both data collection and early stages of analysis were conducted in parallel.^19^ Thus, the emerging findings from the initial analyses informed subsequent data collection and enabled us to probe certain areas. We initially focused broadly on family involvement, with our focus subsequently narrowing as the analysis progressed. Integration of multiple viewpoints and expertise enhanced analytical rigour.^20^ As we had specific research questions regarding how informal carers were involved, including the benefits and challenges and areas for improvement, deductive framework analysis was then conducted on the full dataset using a matrix to facilitate the collaborative team-based approach.^21,22^ The five-stage process outlined by Ritchie & Spencer (1994) was followed.^23^ First, familiarisation with the data was achieved through re-reading the transcripts, field notes and documents included in the initial dataset (L.S.G. and D.L.L.). Second, a thematic framework was defined based on the multi-study research aims (L.S.G. in discussion with E.M. and J.S., and with L.Q., R.T.W. and N.K.). At this point inconsistencies in the data were discussed and opinions on certain aspects of the data and data collection were explored, in addition to the emergent codes. Third, the entire dataset was indexed (coded) by applying the thematic framework (L.S.G., partial coding by D.L.L., E.M., J.S. and S.B.). Fourth, the data were rearranged into a chart to provide an overview of the range of views represented across the dataset in relation to each specific theme (L.S.G.). Finally, the chart was used to map and interpret the dataset by comparing different accounts, identifying similarities, differences and patterns in the data and noting associations between the different themes. Final thematic structure and presentation of results was developed by L.S.G. and D.L.L., and was agreed by all authors. The data were managed using NVivo12 and Excel.

## Results

Patient and carer participant demographics are presented in Table 2; staff participant demographics are presented in Table 3.

**Table 2:** Patient and carer demographics

Participants	Family/carers (total *n* = 4)	Patients (total *n* = 6)
Gender, *n*		
Male	1	2
Female	3	3
Non-binary	0	1
Age, years: *n*		
18–29	0	2
30–49	1	3
50+	3	1
Ethnicity, *n*		
Asian British	1	1
White British	3	5
Employment status, *n*		
Carer	2	1
Student	0	1
Employed part-time	1	1
Employed full-time	0	2
Retired	1	0
Not working	0	1
Patient diagnosis, *n*		
Depression		2
Depression and anxiety		2
Post-traumatic stress disorder		1
Borderline personality disorder		1
Carer relationship to patient, *n*		
Partner/spouse	2	
Parent	2

**Table 3:** Staff demographics

Staff (total *n* = 17)	
Gender, *n*	
Male	6
Female	11
Occupation, *n*	
Mental health nurse/practitioner	12
Support worker	2
Nursing assistant	2
Social worker	1
Median years working in current service	1.5 years
Median years working in mental health	5.5 (range 1–24 years)

**Fig. 1:**
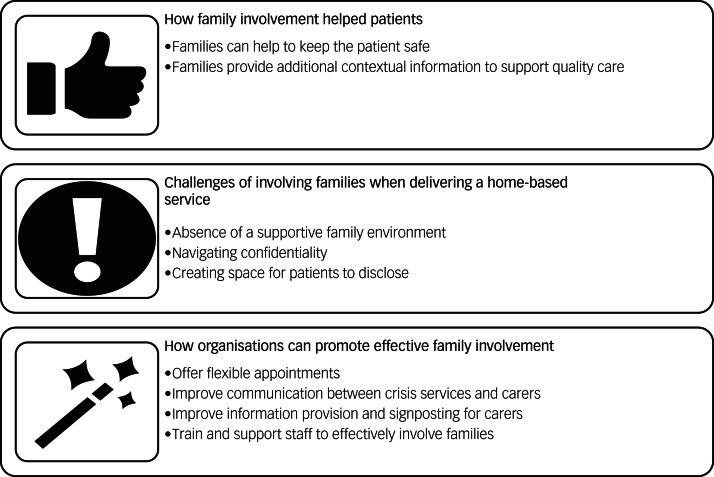
Overview of the three interlinked themes.

Three interlinked superordinate themes, capturing different aspects of family involvement, were developed (Fig. 1): theme 1, how family involvement helps patients; theme 2, challenges of involving families when delivering a home-based service; theme 3, how organisations can promote effective family involvement.

### Theme 1: How family involvement helps patients

The families and friends/carers of individuals experiencing a mental health crisis had a fundamental role to play in the care of the patient. When people were receiving support in their home environment, their family could directly contribute to enhancing their safety and well-being while also providing useful contextual information to healthcare professionals delivering the service.

#### Families can help to keep the patient safe

Where patients were receiving home treatment, appointments with the crisis resolution and home treatment service varied in frequency according to individual need. Outside of these appointments, carers often assumed responsibility for keeping the patient safe from self-harm; for instance, by restricting access to means of self-injury or to medications to prevent self-poisoning, or by providing distraction and support through positive experiences to improve well-being (Table 4, Data extract 1). This was reflected in trust policy documents at the first research site, which stated that ‘The Trust has information leaflets for both service users and their carers on keeping safe that can support the crisis management plan’. Many of the carers and CRHTT staff acknowledged that family members often felt obliged to support the patient, and involvement in care was considered something that carers would naturally do (Data extract 2).

**Table 4:** Theme one: How family involvement helps patients

Subtheme	Data extract number	Quotation
Families can help to keep the patient safe	1	‘boyfriend was able to distract [patient] and support her. He suggested they cook home-made pizzas. which patient said was a good help … Discussed that her boyfriend and family have been encouraging her to do things’ (H6S1 – case notes)
	2	‘I kept hold of the tablets – I had to hide them and all this lot, sometimes you'd forget and then I'd have to hide them again now, and you'd have to find another place’ (F4S1 – carer, partner)
	3	‘We do that quite a lot, advise them [carers] to keep the medication away from the patient and 9 times out of 10 they take control of that medication. And to be fair to the family that's a job that they're doing that we're not. So without the families that patient would be in hospital, so we rely 100% on families sometimes’ (H1S1 – staff)
	4	‘Sometimes she’s a bit, like, when I'm leaving in a morning she's at the top of the stairs as I'm going out of the front door, saying “Are you going to work today? Are you going to work today?” I’m like yeah, where else would I be going? And so she's like “Are you sure?” I'll show you my train tickets when I get back if you don't believe me’ (P1S1 – patient)
Families provide additional contextual information to support quality care	5	‘Just to have them support me and stuff ’cause like they know what’s going on… They kind of like help me to advocate for better care as well, because I kind of like let myself get fobbed off by a lot of different services and I involved them because then they help me get better care for myself’ (P1S2 – patient)
	6	‘Husband reports he has seen improvement with her and she’s more settled’ (P5S1 – case notes)
	7	‘Like you working together, that's why it's good to have good relationships and try and, ’cause when you go and speak to the person and mum’s there, they can help you out by saying well they're not normally like this or they've done this this morning. And say if someone's really depressed and they can't get out of bed to speak to you or something then they can help fill you in’ (H7S1 – staff)

F, family member; H, healthcare professional; P, patient; S1, research site one; S2, research site two.

For the CRHTT staff, having carers actively assisting in keeping the patient safe was fundamental to ensuring that treatment could be carried out in the home when hospital admission would be the only alternative pathway of care (Data extract 3).

However, there was often a fluid and changing dynamic of family relationships that saw greater involvement desired in some aspects of care over others and fluctuation throughout the period of crisis. This made trust and the boundaries of carer involvement difficult to gauge, which in turn placed strain on the carer–patient relationship (Data extract 4).

#### Families provide additional contextual information to support quality care

Outside of physical safety measures, carers also provided an important link between the patient and mental health services, particularly where patients were unable or unwilling to engage. In the case of patient P1S2, their previous negative experience with mental health services influenced their confidence to request the care that they needed. P1S2 reported feeling as though their care was more effective when family were involved (Data extract 5).

During our observations, carers often provided additional contextual information about the patient, to help healthcare professionals understand the difference between how the patient presents when they are well and how they present during crisis (Data extract 6). This was important information for the CRHTT staff to plan treatment, particularly where the patient was unable to provide details. Carers were able to give additional insight into safety and care plans, such as signs of deterioration or improvement in the patient's well-being, and help clinicians to understand the patient's experience (Data extract 7).

### Theme two: Challenges of involving families when delivering a home-based service

Delivering a service in a patient's home presents a series of environmental challenges. These can be particularly pertinent to effective family involvement.

#### Absence of a supportive family environment

Effective involvement of families was not possible in instances where the patient did not have a supportive relationship with their family, or where their family was perceived to be having a negative impact on the patient's well-being and/or mental health. The trust guidelines that we viewed recommend involving families where it is appropriate to do so. This led to individual CRHTT staff deciding whether and how much a particular carer should be involved in a patient's care. Unsurprisingly, the definition of family involvement, and the degree to which it was considered beneficial, varied considerably between patients, carers and staff. Staff were primarily concerned with the impact families can have on the quality of the clinical interaction with the patient (Table 5, Data extract 8).

**Table 5:** Theme two: Challenges of involving families when delivering a home-based service

Subtheme	Data extract number	Quotations
Absence of a supportive family environment	8	‘I don’t know, there's the dysfunctional families, the drug and alcohol situations, what families are up to, are they getting wasted around. There can be a language barrier as well sometimes when English isn't their first language, and mum or dad are trying to interject but there's a language barrier, err, I don't know, I think we do OK really, there are obviously like I say, it's the dynamics when you walk in the room, and then if it's right I'm getting a bad vibe about this or, and then you respond accordingly’ (H3S1 – staff)
Navigating consent	9	‘Husband met us at the door and showed us to the living room where patient and daughter was. Nothing was explicitly discussed about consent for them to be involved and info sharing, but patient probably didn't have much capacity anyway. Also, this was probably dealt with on earlier calls. They are clearly very involved’ (Observation of routine appointment with staff – H1S1)
	10	‘Both yeah, the HTT [home treatment team] asked first and then my mum and dad were like “Yeah, I literally was just going to say that” and then they asked me as well, ’cause first I wasn't actually sure but then if I say yeah then obviously there's gonna be certain things I'm not going to want to talk about and I'm not going to get the right help, so, yeah, the HTT did ask first and then my parents asked me’ (P6S1 – patient)
	11	‘So I suppose for a lot of people it may be that the family are present before they’re even referred to us. For the people that go to A&E [accident and emergency department] a lot of the time they're actually brought there by their family, a lot of the history is taken from the family and then the contact numbers and the details and in terms of how involved they are and the consent gained at that point, so when they then get referred to us we've got a bit of an idea then’ (H6S1 – staff)
Creating space for patients to disclose	12	‘Patient had come to the centre with her boyfriend but chose to come to the consultation alone. Her thoughts changed from not wanting to be here to actual thoughts of killing herself. This was the first time she had experienced these types of thoughts and they distressed her…. We talked about keeping safe and how to distract using mindfulness techniques and activity’ (P6S1 – case notes)
	13	‘Wife answered door and showed us to living room where children was, staff asked if we could talk away from children – which meant that wife could not attend. […] During assessment staff asked if the patient was happy for her to share info with wife – so, if she wants to call the service and ask’ (Observation assessment appointment with staff – H2S1)
	14	‘And I think at the beginning I think it should be, well I don't know if it should be, but I feel it would've been good for us and for her really. Maybe have time out so she can say things without us, which obviously she might not want to say in front of us so that's important but also I think it's important that you can help her say it is, how she's feeling, or give examples of situations that’ (F6S1 – carer, mother)
	15	‘When I’ve been assessed like alone in the crisis team it’s resulted in a better outcome because I’m like more honest and I think sometimes like my mum might take that the wrong way sometimes coming from me so it’s better to come from the staff I feel like. Then it doesn't put the pressure on me to be like ‘‘oh can you not be here for this’ (P1S2 – patient)

F, family member; H, healthcare professional; P, patient; S1, research site one; S2, research site two.

Working with families was a skill that staff reported developing through experience rather than something that is taught. On the whole, staff spoke about having the confidence to ask challenging family members to leave, or finding workable solutions to known problematic family situations: for example, ‘I'd maybe take a colleague and do it in twos so somebody can do the assessment and someone can manage them’ (H3S1).

#### Navigating consent

Consent to having carers in attendance during clinical reviews was often implied by the family being present in the home when the CRHTT staff arrived. In our observational data, family members or carers often welcomed CRHTT staff into the home and led them to the room where the patient was waiting. Observational data showed that gaining consent to involve carers in clinical interactions or to share information with them was inconsistent and absent from extracted case notes (Data extract 9). Instances of good practice showed where practitioners sought patient consent to involve carers at the start of each review (Data extract 10).

However, with some patients who were known to the CRHTT or were referred from a hospital emergency department, consent for the carer to be present in clinical reviews was often assumed (Data extract 11).

#### Creating space for patients to disclose

Throughout the course of care observed, involvement of carers changed across clinical interactions. When carers were not present this afforded the patient space to disclose thoughts of suicide or self-harm (Data extract 12). In addition, creating space for disclosure enabled patients to discuss their family context and to raise any issues where family members may have negatively contributed to the patient's mental health and well-being. However, it was clear during observations that creating space for disclosure can be challenging, given that the home environment was often a space shared with partners, parents and children (Data extract 13). Family and carers recognised the importance of patient confidentiality and for the patient to have space for disclosure (Data extract 14).

Many of the patient participants were concerned with balancing carer involvement with exposing them to potentially distressing information. Asking carers to leave could be challenging for the patient to navigate. The carers interviewed for this study appeared to understand the patient's need for space. However, practitioner participants reported previous examples of where they have had to ask carers to leave a review. All staff participants routinely tried to create space for the patient to attend at least part of the review alone (Data extract 15).

During observations, we visited homes that did not offer a truly private space to conduct the session. Both services were able to offer a private meeting room at one of the trust's service locations as an alternative. However, only one of the services appeared to routinely offer this as an option for patients.

### Theme three: How organisations can promote effective family involvement

At an organisational level, home treatment teams can make adjustments to service design and delivery to increase the likelihood of effective family involvement.

#### Offer flexible appointments

One of the main barriers to effectively involving carers in a patient's mental healthcare was the ability of carers to attend appointments. In one trust, appointments were often not scheduled for a particular time, but instead a rough approximation was given. Acknowledging the importance of family involvement to patient care lay in tension with the reality of many carers’ lives. Some carers had young children, which prevented their involvement in their partner's session. For carers who worked, attending appointments proved particularly difficult (Table 6, Data extract 16).

**Table 6:** Theme three: How organisations can promote effective family involvement

Subtheme	Data extract number	Quotations
Offering flexible appointments	16	‘You’ve got family members who’ve got to take time off work to support individuals who have struggled with crisis and obviously you’ve got the loss of income from that then and it’s like a snowball effect with some people isn’t it, like family involvement, it's what their involvement is, the knock on effect of that, you know’ (H4S2 – staff)
	17	‘I mean I wouldn’t necessarily change the times of the visit so that the family could be present because of the nature of what we’re doing and the risk for that patient but I would certainly speak to them’ (H1S1 – staff)
Improving communication between crisis services and carers	18	‘Even when the lady came I thought she might speak to me, but she didn't speak to me. She just saw my husband, then she left. [Interviewer: “OK, do you know if they left any information about if you needed to get back in touch with them at all or…”] I don’t know if they have left it with [patient] but I didn't see any. He told me that “I am discharged now”, he only told me’ (F2S1 – carer, partner)
	19	‘I felt shut out actually and I didn’t know what was going on and even though it was so hard to hear what she was saying and it was upsetting, I was aware of what she was thinking whereas I didn't know what she was thinking when she went on her own, do you know what I mean’ (F2S2 – carer, mother)
	20	‘Yeah, I mean like they do say to call the urgent care hub, but from like my experience they don't take referrals from family members, like they kind of talk to them on the phone and say like we're not taking referrals until we speak to the patient and get their consent, but then it adds to anxiety on discharge – if something were to happen what would I do then?’ (H5S2 – staff)
	21	‘Just a bit more how we could support her more, in whatever way that is, you know. Because if I saw her a bit quiet, I would talk more, but sometimes you just need not to talk as well to her, so there's that as well but it's just, for us to help her cope and how to help her, you know we can think ourselves a little bit, but are we saying the right things because I think that's the most important thing, because I know I wouldn't say anything negative to her, because I know that wouldn't be what you should do, but it's what, how or even how to say or what to say that would be best for her, that's what I felt. We were struggling with things of how we can do it’ (F6S1 – carer, mother)
	22	‘If I hadn’t have been with my family or if I hadn’t been mentally where I am at the moment then do I think my chances are of still being here, I doubt it. You know what, that would have been blood on my hands. I've always said I take responsibility for things but I don't take responsibility for a system that is just, there are so many people who have lost their lives and even [nurse] said it, she said “I've seen people fall through these cracks” – it's not even a crack. There's no net or anything’ (P2S2 – patient)
	23	‘I’ve noticed a lot more calls from family members actually ringing us for updates or saying “Can I discuss this with you?” or “Can you make a note of this?” and I don't think there's been prior to COVID that many calls from family members trying to actively link in with us in between visits’ (H2S2 – staff)
Improve information provision and signposting for carers	24	‘When I started in crisis resolution and home treatment [CRHT] I had absolutely no idea on what services and our role is signposting on to other services because our main role is prevention, um, so actually when I first started I took time in my day to research what services were near me and to find out about the befriending service, and [name] and some of the service users I come across will tell me about groups they go to and I try to keep that information. Some information has come through other colleagues that have been community-based for 20 years and I have a little app on my phone which I share with all the service users which tells you about what's on in the local area’ (H7S2 – staff)
	25	‘They left me a leaflet to go to these once every few weeks on a Wednesday. I can't remember what it's called now, it's just for people, like the partners, who have been through or going through. It just gives you a bit of an hour, you know, coffee, to speak to some other people’ (F4S1 – carer, partner)
	26	‘I would tend to just go online and find some easy-to-understand information, ’cause everybody Googles don't they, and it makes it worse ’cause then I'll get a family member who'll say “I looked online last night about this and have they got that as well, why you giving them that medication?”’ (H1S1 – staff)
Train and support staff to effectively involve families	27	‘I think it’s harder if there’s conflict and they’re saying “Don’t tell my mum about this”, so I think there is always benefit in training around conflict in families and confidentiality because it's a minefield isn't it? I suppose it would be helpful to have an idea how to manage that and what solutions can be found because to be honest I think most of us, like, wing it’ (H3S2 – staff)
	28	‘It’s kind of talked about as a bit of a buzz word isn’t it, like family involvement and carer involvement, but I don't feel like there's ever any actual training in it, you just like learn it on the job. I don't think there's any specific training on, like, when it's a good idea to, when it's not a good idea to engage people, you know, family members and stuff. I remember when I went on the ward I was like a champion for carers like. [I] had only been qualified about 6 months. It was just like they need a name to put on the board for like the champion – you didn't have any specific training or anything’ (H5S2 – staff)
	29	‘A monthly meeting where the community staff get together with a psychologist, because that used to be really beneficial because you'd come away from that meeting and go, yeah, next time she does that I'm not going to react the way I did because we're all human aren't we?… Well, actually being able to break it down and understand why they're doing it, you then respond to them better’ (H1S1 – staff)

F, family member; H, healthcare professional; P, patient; S1, research site one; S2, research site two.

To counter this, the services we observed offered visits outside of traditional working hours, and some staff advocated maintaining flexible appointments to enable carers to attend reviews. Where a mutually suitable time could not be found, it was expected that adequate communication, such as telephone calls with involved carers, would occur outside of the scheduled appointment times (Data extract 17).

#### Improve communication between crisis services and carers

Enhancing this communication was crucial to enabling carers to feel involved in the care of patients. Carers spoke of relying on patients to inform them of developments in care plans, where there was little communication or sharing of safety and care plans between practitioners and carers. For carer F2S1, this lack of communication between the CRHTT and carer meant that they learned of the patient's discharge from the patient (Data extract 18).

Although acknowledging the need for patient confidentiality during appointments, carers also reported feeling excluded from important information, which affected their ability to support the patient and keep them safe. Carer F2S2 spoke of ‘feeling left in the dark’ when the consent for their involvement changed and they were no longer included in patient reviews but received inadequate follow-up communication or explanation as to why consent had changed. During observations, the patient had requested space to discuss with the CRHTT important parts of their story and make sense of their relationships, and felt that this would be more beneficial without family present. Without this understanding, the carer felt that they might have been missing important information on how to keep the patient safe (Data extract 19).

Staff reported encouraging carers to contact the CRHTT with questions about the patient's care or the urgent care hub for post-discharge concerns. However, clear barriers existed to carers seeking support from available services (Data extract 20).

Where staff reported encouraging carers to call the CRHTT to discuss any concerns, carers said they would prefer to receive more detailed information during debrief conversations. Carers recommended that debriefs should focus on how to support the patient appropriately and keep them safe at home. With lack of direction, carers often felt anxious to know that they were saying and doing the right thing for the patient (Data extract 21).

Communication between staff, patients and carers was particularly important during discharge from the CRHTT, when there are often long waiting times for post-discharge services. As P2S2 describes, an unexpectedly long wait for an appointment with a follow-on service meant that they became increasingly reliant on family to help keep them safe, adding to the existing burden of responsibility that carers experience (Data extract 22).

During the COVID-19 pandemic, the inclusion of carers became more challenging as restrictions on inter-household mixing and an initial move to remote consultations meant that some carers were unable to participate meaningfully in appointments. This was acknowledged as unusually challenging for the crisis service, although debriefing absent carers was essential to giving the best quality of care for the patient. Staff reported being keen to quickly resume face-to-face patient contact. Services noted that telephone communication with carers increased in the absence of in-person visits (Data extract 23).

#### Improve information provision and signposting for carers

Information provision and signposting for carers was inconsistent across both research sites. Although many practitioners identify that part of their role is to support the family, many do not utilise their NHS trust's carers’ support service, instead seeking local carer information on an individual basis. Many practitioners identify local carer and patient (service user) groups, or rely on colleagues or other patients for information (Data extract 24).

Carers welcomed information about carers’ support groups that could help them gain peer support throughout the patient's mental health crisis. Many of the carer participants spoke of feeling isolated, with access to a carers group providing the opportunity to receive support and also to learn from others about how best to support the patient (Data extract 25).

Where carers were not provided with additional information, they resorted to searching the internet to find the information that they needed. Lack of access to appropriate information presented a challenge as staff would need to spend additional time explaining care strategies. Some practitioners reported providing carers with standard information to increase understanding (Data extract 26).

#### Train and support staff to effectively involve families

Trust policies included the provision of training in working with carers/families. However, all staff in this study identified family and carer involvement as an area in which they received little or no formal training, but felt that trust training or shared learning would be of benefit to their individual practice. Staff were keen for training to be scenario-based face-to-face shared learning rather than mandatory e-learning. They suggested that any further training should give guidance about what to expect when working with families, how to identify and engage carers, what questions to ask and how to ask them, and how to navigate conflict and confidentiality (Data extract 27).

For practitioner H5S2, previous experience of being a named carer's champion was felt to be a tokenistic nod to good practice rather than being embedded in good practice (Data extract 28).

Practitioners felt that having access to clinical psychology services for discussion of family involvement may be beneficial. Practitioners envisage the opportunity to reflect on challenging experiences with patients and carers, to better understand carers’ needs and to be supported in understanding why such experiences occur and how they can improve their responses in the future (Data extract 29).

## Discussion

Findings from this investigation enhance our understanding of how family involvement may contribute to improved patient safety and well-being for individuals accessing acute home-based mental health treatment. The triangulation of viewpoints and utilisation of different data collection methods yielded novel insight by describing: (a) how family involvement can help patients, (b) the key challenges that may prevent effective involvement and (c) what organisational adjustments can be made to promote effective involvement (summarised in the Appendix below). This study was carried out before and during the COVID-19 pandemic, when the impression was of services placed under constant intense pressure, resulting in increased case-loads and short-term intervention.

Participants identified family and carers as having an active role in keeping patients safe, supporting them in activities to promote improved well-being and providing additional contextual information during clinical interactions. However, we found key challenges to involving family members effectively when delivering a home-based mental health service. Specifically, these were the ability to ensure a patient's right to confidentiality and the provision of the opportunity for patients to disclose sensitive information confidentially. Additionally, staff training in how to work with families was lacking but considered a beneficial point of learning by staff. Healthcare professionals identified areas for shared learning to improve care for both supportive and challenging families. Further, it was acknowledged that there may be instances when effective family involvement may not be feasible owing to the absence of supportive, healthy family–patient relationships. It follows that absence of supportive family involvement may affect the level of support that is consequently provided by the CRHTT. The key implications for healthcare providers are to design services that can offer flexible appointment times outside of traditional working hours and to routinely offer an alternative space for sessions, particularly for those for whom a truly private space does not exist in the home. In addition, carers felt that improvements could be made in communication and dissemination of information, including safety plans, care plans, carer groups and support. Healthcare professionals identified areas for shared learning to improve how services work with both supportive and challenging families.

### Comparisons with existing literature

Consistent with those reported from previous research, our findings suggest that actively involving families and carers in a patient's mental healthcare may contribute to improved outcomes.^7,8,23–26^ Despite national guidelines recommending the involvement of the family or carers, routine implementation is inconsistent.^6,13,14,27,28^ Our findings suggest that this may in part be due to challenges in two key areas.

First, we discovered that healthcare professionals held varying beliefs on the extent to which they should involve families in care and this may explain some of this inconsistency at local level, although all agreed that family and carers can play a key role in patient safety. Wyder et al (2020) noted that there is limited literature to guide how healthcare systems can assist staff in supporting patients.^11^ Echoing Berzins et al (2018), some concerns remain about whether patients and carers feel that their worries regarding patient safety are heard by services, with some participants reporting a desire for knowledge on optimal approaches to patient safety and searching online for information, where the preference would be for this to come through the service.^29^

Second, navigating privacy and confidentiality to satisfy the different needs of patients, family members (and other carers) and staff is a known challenge in acute mental healthcare.^7,15^ The current study highlights the additional logistical challenges posed when delivering support in patients’ home environments. Some carers in this study acknowledged the need to allow patients to engage in clinical interactions without family present, echoing findings by Slade et al (2007) and Soklaridis et al (2019).^30,31^ This may be more challenging in smaller homes or those with lots of occupants. Further, Landeweer et al (2017) suggest that family members may worry that their involvement could have a negative effect on the patient–clinician relationship.^15^ During data collection in some homes, we observed the lack of truly private space when delivering sessions, which was a challenge for staff, patient, carers and their families.

We found that the provision of written information and communication of updates regarding care following clinical interactions was lacking or inconsistent across families and trusts. Enhancing communication may help to develop better partnerships between services, patients and carers, and may be particularly supportive for both patients and carers following discharge.^32^ Indeed, Haselden et al (2019) showed that where families are involved in patient care, patients received more comprehensive discharge planning and their attendance at follow-on out-patient appointments was more frequent.^8^ The involvement of families may be more important at discharge from crisis services, as there may be long waiting times or gaps between services for ongoing patient care. Indeed, direct continuity of care (via telephone or face-to-face contact) is needed to reduce post-discharge suicide risk.^33,34^

### Strengths and limitations

Utilising an ethnographic approach yielded detailed observational and interview data, enabling access to practices, habits, behaviours and views that would not normally be learned about by other means. The inclusion of multiple viewpoints from patients, carers and healthcare professionals is the study's main strength. Additionally, the research team's multidisciplinary composition, including psychiatry, health services research, qualitative expertise and patient and public involvement, enabled interpretation from multiple perspectives.

However, the reported findings should also be considered in relation to the study's limitations. Recruitment at the second research site was hindered by the COVID-19 pandemic, which prevented the recruitment of a larger and more diverse participant sample, as was originally planned. The participants represented only a minority of cases at each research site, but we had good coverage of the presented themes within our data. Furthermore, as this was not a consecutive case series design, we relied on staff to identify appropriate patients to approach. This could have naturally led to some bias in recruitment and reduction in the variation of potential participants; for instance, as both researchers were female, staff did not approach patients who were only to be seen by male CRHTT staff. At the second research site, this restriction also extended to patients with drug and alcohol use disorders. At the second site three potential patient participants and two carers chose not to participate in the study, although their reasons for declining were not recorded. Staff identifying potential participants might have influenced whether the patient decided to participate and how they reported their experience of family and carer involvement. To address this we aimed to maintain privacy as much as possible by conducting individual interviews, giving participants the space to share their thoughts without staff or family present. Transcription was carried out by the researcher who conducted the interview and all data were pseudonymised to protect the participants’ identity.

Owing to research restrictions related to the COVID-19 pandemic, observations of clinical practice at the second research site could not include data collection at all points on the care pathway that ideally we would have secured. Furthermore, reviews of clinical notes were restricted as this was reliant on access by healthcare staff who were largely working remotely. Extracted clinical notes and observational field notes that lacked sufficient depth or risked breaching the participants’ anonymity were excluded from use as illustrative data extracts.

Although our findings suggest that effective family involvement was perceived to contribute to improved patient well-being and safety, it is important to note that we did not examine quantitative indicators of beneficial outcome such as improved patient scores on clinical tools or reduced incidence of self-harm.

Future research should explore the impact of social positionality and sociodemographic variables on the relationships between patients, carers and mental healthcare professionals at different points in the treatment pathway.

### Clinical implications

Our findings indicate that healthcare professionals must initiate explicit discussions about consent and carer involvement, thereby enabling carers to be present during clinical interactions and to open and maintain communication pathways with involved carers. The involvement of carers is particularly important when planning to discharge a patient from crisis services. Understanding the positive impact of open, honest communication within families and wider support systems highlights the importance of establishing this approach from initial assessment. It is encouraging that some of the practices revealed in this study concur with evidence-based recommendations from the National Confidential Inquiry into Suicide and Safety in Mental Health and National Institute for Health and Care Excellence (NICE) clinical guidelines and with local NHS trust policy on involving families. However, further training and shared learning is required, particularly regarding navigating confidentiality and privacy in a home environment, as specified in an information-sharing and suicide prevention consensus statement published by the Department of Health and Social Care,^35^ and ensuring regular and timely communication between families and services. Insufficient resources and current service demands may, however, hinder implementation. These findings highlight that enhanced family involvement in mental healthcare would likely have benefits for every party in the triangle of care.

## Data Availability

De-identified interview transcripts that support the findings will be available via the UK Data Service (https://ukdataservice.ac.uk/) depending on informed consent of individual participants. Observational data are not publicly available because they contain information that could compromise the privacy of research participants.

## Appendix

Service considerations for improving family and carer involvement:

- improve family and carer involvement
- standardise the identification of family members as carers and signpost to trust and local carer services
- create opportunities for shared learning on working with families for crisis resolution and home treatment teams (CRHTTs)
- improve communication between CRHTTs and carers – with conversations and evidence-based learning, including de-escalation, how to keep the patient safe and how to support them, in addition to discussion about changes in risk/severity of illness
- improve sharing of care plans and safety information between CRHTTs and carers
- consider offering flexible appointment times outside of traditional working hours and the option of alternative spaces, should it be not possible to ensure privacy within the home environment.
